# Topics of Public Interest

**Published:** 1918-10

**Authors:** 


### TOPICS OF PUBLIC INTEREST

---

**German Births** were reduced from 1,839,000 in 1913 to 1,103,000 in 1917, respectively 27 and 16.5 per 1000 of the antebellum population of 67,000,000. As we have suggested, it is not improbable that the war will deplete the European population even more in the young and old than in the men of military age.

---

**It is Estimated that 7,600,000 Gallons of Gas** will be saved each Sunday while the ban on Sunday auto riding continues.

---

**Major Charles A. Reed,** M. R. C., conducted a short campaign for enlistment of doctors in the Medical Reserve Corps for Erie County. Erie County has been 69 below its quota but as the results of the lectures 65 physicians have qualified and now await call.

---

**The War Department** states that of the American soldiers wounded in the Marne-Aisne offensive and sent to hospitals, 1 in 20 die. That of all the soldiers sent to the hospitals only 45 in every 1,000 die. That more than four-fifths return to service. That 14.5 per cent are discharged for disability.

---

**Army Surgeons** have discovered a means to combat Empyema, a strange disease of the lungs that assumed the proportions of an epidemic in many cantonments last winter.

The form of the disease that appeared in the camps last winter differed from the ordinary disease seen in civil life, being due to the streptococcus, one of the most dreaded germs known.

Empyema is an infection that attacks the membrane covering the lungs or the membranous lining of the chest walls. It produces a fluid, oftentimes pus, which remains between the two membranes. The amount of the exudate constantly increases and the lungs are forced up into a smaller and smaller space as the fluid tends to fill the pleural cavity. During this stage of the disease breathing becomes more labored and difficult, due to the restricted air space in the lungs. Aside from this aspect of empyema, which alone may prove fatal, the virulence of the germ is such that the infection is always to be dreaded.

While similar epidemics have ravaged all armies for the last three centuries, at no time has the cause been known. The first known operation for empyema was performed in a similar epidemic during our War of 1812.

---

**A Health Rate,** which as far as known has never been surpassed, has been established by the American Armies both here and overseas. For the week ended July 26 the combined reports of the American Expeditionary Forces and of troops stationed in the United States show an annual death rate of disease of 1.9 per 1,000—less than 2 men per 1,000 per year. The annual death rate for disease of men of military age in civil life is 6.7 per 1,000. This new rate is based on an approximate strength of 2,500,000 men, and includes men living under abnormal conditions.

---

**Surgeon General Gorgas** has called for 1,000 graduate nurses a week—8,000 by October 1. 25,000 graduate nurses must be in war service by January 1—in the Army Nurse Corps, in the Navy Nurse Corps, in the U. S. Public Health Service in Red Cross war nursing. This involves withdrawal of many nurses from civilian practice and necessitates strict economy in the use of all who remain in the communities. You can help get these nurses for our sick and wounded by: Relieving nurses where possible wholly or in part from office duty. Seeing to it that nurses are employed only in cases requiring skilled attendance. Insisting that nurses be released as soon as need for their professional service is ended. Seeing that your patients use hospitals instead of monopolizing the entire time of a single nurse.

---

**A Five Million Army Means Fifty Thousand Medical Officers.** With an army of three million men in the field or in training and as contemplated, an expansion of this force to five million men, the Surgeon General must have in the Medical Reserve Corps at least fifty thousand doctors.

The Medical Corps must keep a pace in growth with the army expansion and it behooves every doctor in the United States between the age of 21 and 55, who is physically morally and professionally fitted, to arrange at the earliest possible moment, his personal affairs so as to offer his services to his country in the capacity of a medical officer.

The United States is in the war to do her part in winning the struggle and this can only be accomplished by a large and well trained body of troops adequately cared for by sufficient number of medical officers. The importance of the doctor's service and its relation to the successful outcome of the war cannot be under-estimated.

As the mobile forces increase in size, so is there an expansion of Base Hospitals and other Institutions for the care of the sick and wounded and there should be no lack of officers when required to give to our patriotic boys, the professional attention which is so essential.

It is well for the medical profession of the United States to realize at once that a Medical Reserve Corps of at least 50,000 doctors will be required to meet the demands of the Surgeon General and upon which Corps he can draw for his medical officers.

We believe by this time that the profession of this country must be fully alive to the needs of the Service, so let every doctor who is qualified, feel that he is doing not only his patriotic duty in offering his services as a medical officer, but is relieving the tension of the Surgeon General's Office by placing at the command of the Chief Officer of the Medical Department an adequate force without the frequent beating of drums to supply the necessary number with each increase of the mobile forces.

If you have not already received an application blank for commission in the Medical Reserve Corps, your nearest Examination Board or the Editor of this journal will be glad to supply you.

---

**Miss Anna G. McCrady** and five other nurses of the Ft. Porter Base Hospital left for overseas service about Aug. 1, forming part of a psychiatric unit of 40. News of this movement was withheld till too late for our Sept. issue.

---

**Volunteers for the M. R. C. Still Needed.** The announcement that no further candidates for officers' training camps would be taken from civil life has, very naturally, been understood to apply to all. As a matter of fact volunteers for medical commissions are not only acceptable but are urgently needed to make up the average of 1% of the total number of troops. At least 5,000 physicians are required for forces already inducted or who will be in the very near future. The editor can personally vouch for the fact that averages of 50 physical examinations or of 100 patients per physician are maintained almost constantly at various places. Medical military work can not be evenly adjusted but there is no favoritism in the distribution of hard assignments. If anything, the hardest work is done by the members of the regular army medical corps and by those of the higher ranks. The medical profession should appreciate the fact that the only way in which the overwork of their military confreres can be reduced is by a generous response to the call for more volunteers.

---

**Automobiles** numbered 404,247 for the state, 4,810,917 for the country in 1917; 421,084 for the state, 5,461,791 for the country on June 1, 1918. The average increase was 15%, the percentages varying so as to show that certain states are very close to the necessary supply, while others are increasing rapidly up to approximately the same per capita basis. For example, Massachusetts increased only 1%, N. Y. 4%, while F. Va. and N. J. increased 47%.

---

**The War Department** announces that a gold chevron of standard material and design will be worn on the lower half of the left sleeve of all uniform coats, except fatigue coats, by each officer, field clerk, and enlisted man who has served six months in a theater of operations during the present war as an officer, field clerk, or enlisted man of the armies of the United States, and an additional gold chevron for each six months of similar service thereafter. And that a gold chevron of pattern identical with that of the war service chevron, to be worn on the lower half of the right sleeve of all uniform coats for wounds received. Disablement by gas necessitating treatment by a medical officer shall be considered to be a wound within the meaning of this order.

---

**Sugar.** Since the Government has been handling the purchase of sugar, through the United States Food Administration (Sept. 1, 1917), approximately 200,000,000 pounds have been used by the Army. This amount is exclusive of the depots and camps on the western coast, which have been using raw sugar from Manila, having it refined in the West. A conservative statement of the amount of sugar procured on the Pacific coast is about 25,000,000 pounds, making the total purchase for the Army 225,000,000 pounds. It is found that about 237 pounds of sugar are consumed by 1,000 men at their meals in one day.

---

**Chiropodists.** As far as is consistent with the military demands, chiropodists taken into the Army will be transferred to the Medical Department and either assigned directly to the various camps for duty under the camp surgeon or first sent to Camp Greenleaf for further training under the regular orthopedic instructors. On the demonstration of proper skill and attainments they may be advanced to the grade of sergeant. A canvass of the camps is now being made to determine the need of this particular service.

---

**Nutrition Officers** are to be stationed in every National Army cantonment and every National Guard camp, as well as in every camp where 10,000 or more soldiers are in training. These officers are food specialists who before they joined the Army as members of the Medical Department were connected with colleges and public bodies as physiologists, chemists, economists, food inspectors, and experts in other specialized work relating to food.

---

**Corps of Doctors and Trainers.** For the care and conditioning of fliers in the Air Service the United States Government is now appointing a corps of doctors and trainers large enough to equip each training field and camp for fliers, both here in the United States and in France, with a proper organization. The doctors will be known as flight surgeons and the trainers as physical directors. The medical branch of the Air Service is not alone confined to the selection of the flier but to his care and condition after he has been admitted to the service.

---

**Government Insurance.** More than $30,000,000,000 of Government insurance has been written to date to protect America's fighting forces and their families, Secretary Mc-

Adoo announces. Approximately 3,400,000 insurance applications have been received by the Bureau of War-Risk Insurance of the Treasury Department up to the close of business August 30.

---

**Provision for Wounded From Europe.** Debarcation and other hospitals have been provided to accommodate 90,000, with increase to 100,000 by slight crowding. As a quarter of casualties consist of deaths and as somewhere near 80% of the wounded may be expected to return to the fighting line after treatment in overseas hospitals, these numbers correspond to a total casualty list of about 1½ million, 50 times the aggregate at the end of the first week of Sept.

---

**French Military Statistics.** It is estimated that France has lost a million soldiers by death and another million by permanent crippling, there remaining a force of 4¾ million. Typhoid morbidity has fallen from 7.:1000 in Dec. 1914 and Jan. 1915, to 0.1-0.06:1000. The annual mortality rate from typhoid has fallen from 98:100,000 in 1914 to 0.3 in 1917. Venereal admissions have fallen from 21 to 14:1000. The mortality of the wounded in the recent Aisne offensive was 5.18% although 20% were considered too severely wounded to be transported to the base.

---

**American Casualties.** The total casualties of the American Expeditionary Force to Sept. 7 were just over 30,000, about what the British army of about 2 million has suffered per week on the average, since the beginning of the German drive in March. In some respects, the distribution of casualties is considerably different from what might be expected, from present experience in other forces and from past experience in general. We have previously called attention to the fact that while in all the wars of history up to the Russo-Japanese War, deaths from disease have exceeded those from fighting, the ratio being about 2:1 for the Civil War and as high as 10:1 in many campaigns of earlier date, all the enlightened nations of the present conflict have maintained a disease mortality if anything less than what would be expected in peace for the same adult male class. Our own deaths from this cause are only 325. Owing to the gradually increasing numbers of the force and the different periods for which each increment has been a part of the force, it is impossible to calculate a death rate. Accidents, inevitable to and really forming an indirect item of military losses, have caused 789 deaths. The actual military fatalities, no attempt being made to differentiate between immediate killing and deaths from wounds, total 7,662, almost exactly 24.5% of the casualties.

Missing in action, including prisoners and unnoted deaths and some others, number 3,424. These statistics show definitive losses, up to delayed deaths from wounds and permanent military disability, of 40% which is about the average of all definitive losses according to recent military experience. The high and low claims by other countries of return of wounded to duty, 90-80%, would make our definitive losses, respectively slightly under and slightly over 50% of the total casualties. Most of those whom we have asked as to an explanation of this high percentage of definitive losses have said that it was due to the lack of caution of inexperienced troops. But imprudence would increase the number of missing, especially prisoners and, by the law of chance, would increase the number of slight wounds at least as much as the fatal ones. The only explanation that occurs to us is that American soldiers continue fighting after receiving their first wounds, until they are killed or more severely wounded.

---

**National Taxes**, will amount to nearly $80 per capita in the present year of the war, beside about $200 per capita to be raised from bonds and war savings stamps. Ordinarily, the national government spends about $10 per capita.

---

**Opening for Nurses in State Hospitals.** About 6000 nurses are employed in the State (Insane) Hospitals of N. Y., to care for about 37,000 patients. Many nurses have entered the government service and it is possible that the clientele of the hospitals may be increased by the admission of patients from the army, as all citizens of the state are to be admitted from military and naval service, if so desired by the military authorities. Hence the State Hospital Commission has provided for the admission of 1000 young women to state hospitals for training from permanent positions. Application should be made to the Commission at Albany. Male students exempt from military service will also be taken. Perquisites and salaries will render the students self-supporting.

---

**Economizing Nurses' Services.** The Buffalo District Nursing Association will hereafter, limit the duties of its nurses to strictly professional care, such services as washing and combing hair, arranging trays, etc., being left to members of the family, subject to instruction. The aim is to release more nurses for military service.

---

**British Physicians** living in the U. S. are urgently called by the Canadian government to volunteer for service. Degrees from medical schools of the United States will be honored and commissions will be issued immediately. Those in this locality should apply to Capt. A. Cunningham Tweedie, 333 Main St., Buffalo.

---

**Ambulance Wrecked.** The Buffalo Homoeopathic Hospital ambulance was wrecked in a collision with an automobile Sept. 7. No one was injured.

---

**Commissions for Women Physicians.** Of interest to all women of this country is the news that three American women physicians have received lieutenants' commissions in the French army and are the first American women to obtain army rank. They are Dr. Caroline Finley, Dr. Anna von Sholly and Dr. Mary Lee Edward, all of New York, and attached as surgeons to the military unit of the women's oversea hospitals, financed and managed by the National American Women Suffrage Association. The women received decorations from the French government for excellent surgical work recently performed under heavy bombardment, and after that, their commissions.

---

**Stenographers and Typewriters Wanted.** The U. S. Government still needs many workers in these lines. Examinations are held every Tuesday in 550 cities. Most of the employment will be in Washington. The government has taken steps to remedy the high charges for room and board by listing boarding houses and building residence halls with cafetarias. Two persons rooming together can secure room and two meals a day for about $40 a month. The pay is liberal. For further information address John A. McIlhenny, President U. S. Civil Service Commission, Washington, D. C.

---

**Antiseptic Formula for Gas Bacillus Infection.** Maj. James T. Pilcher, of Brooklyn, now at a base hospital at Auteuil near Paris, has had excellent results from the following, used exactly like Dakin solution. Quinine sulphate 1, HCl 0.5, Glacial acetic acid 5, NoCl 17.50, Thymol 0.25, Formol 1, Alcohol 15, Water up to 1000. The quinine is dissolved in the two acids, the thymol in the alcohol, the salt and formol in the water, before mixing. The solution is practically permanent, has been tested in the worst possible cases of comminuted fracture, and has superseded the less permanent Dakin solution in this hospital.

---

**Spanish Influenza.** It is reported that there were on September 25, 29,002 cases of so-called Spanish influenza in various army camps. Fifteen camps and stations had no cases.

